# Supplementary material for: Extensive gut virome variation and its associations with host and environmental factors in a population-level cohort
Source: Nat Commun. 2022 Sep 6;13:5252. doi: 10.1038/s41467-022-32832-w (PMC9448778; doi:10.1038/s41467-022-32832-w)
Supplement: Supplementary file 1 — Supplementary information [file 41467_2022_32832_MOESM1_ESM.pdf]

Supplementary information for

Extensive gut virome variation and its associations with host and environmental factors in a population-level cohort

Suguru Nishijima*, Naoyoshi Nagata*, Yuya Kiguchi, Yasushi Kojima, Tohru Miyoshi-Akiyama, Moto Kimura, Mitsuru Ohsugi, Kohjiro Ueki, Shinichi Oka, Masashi Mizokami, Takao Itoi, Takashi Kawai, Naomi Uemura, & Masahira Hattori

*Corresponding author:
nnagata_ncgm@yahoo.co.jp (N.N.); nishijima.suguru@gmail.com (S.N.)

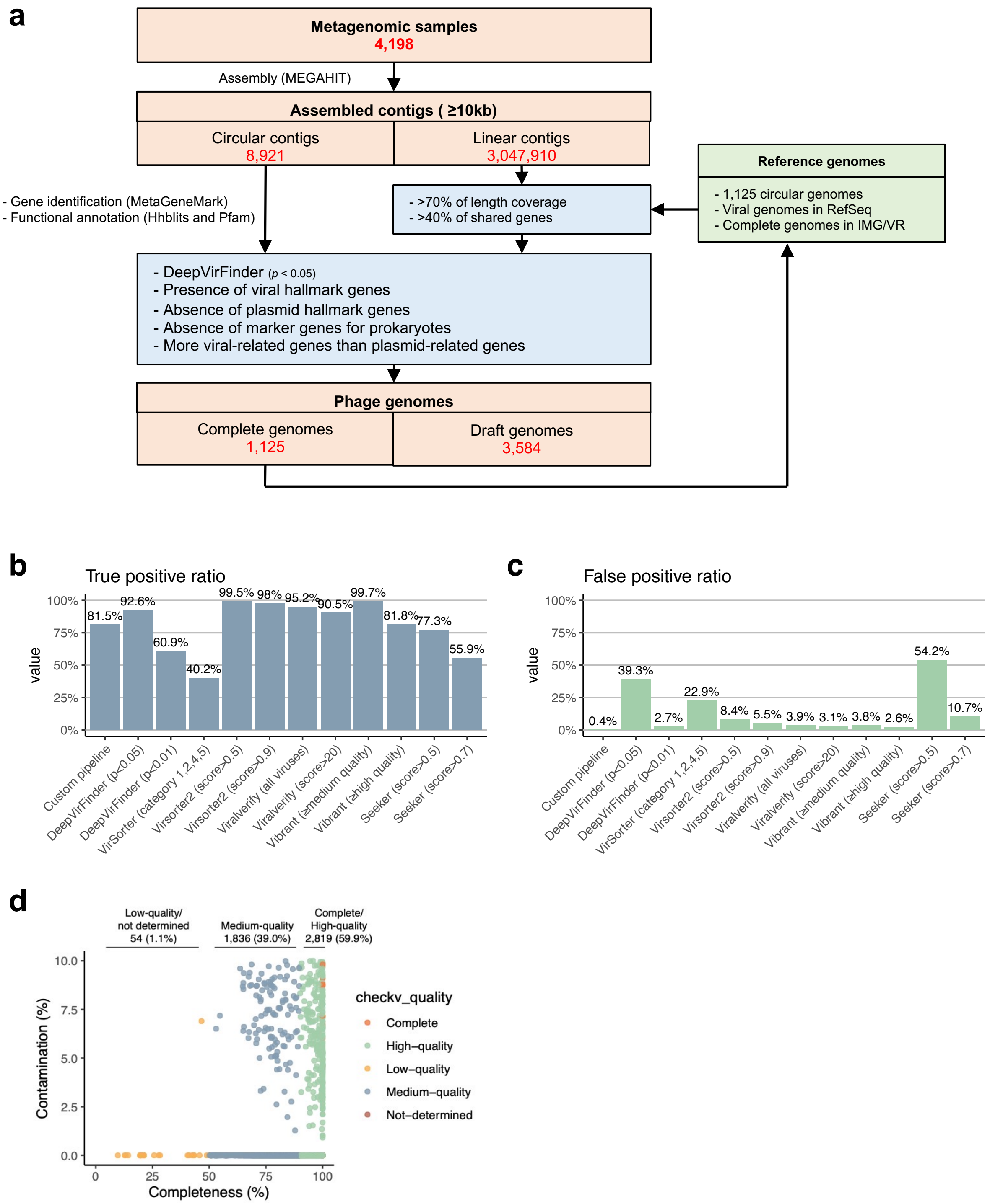

Supplementary fig. 1 | Overview of the custom pipeline for phage detection and a comparison of its performance

a, Workflow of the phage-detection pipeline. Details of the pipeline are described in **Methods**. **b, c**, Comparison of the performance of the virus detection pipeline. The performance of the pipeline was evaluated using reference phage genomes (n = 2,609) as positive data and plasmids (n = 16,136) as negative data, downloaded from the RefSeq database. The true positive ratio was defined as the number of phages detected as phages divided by the number of total reference phages. The false positive ratio was defined as the number of plasmids detected as phages divided by the number of total reference plasmids. **d**, Quality assessment of the phage genomes. The 4,709 phage genomes obtained from the 4,198 metagenomic data were evaluated by checkV and their completeness and contamination were estimated.

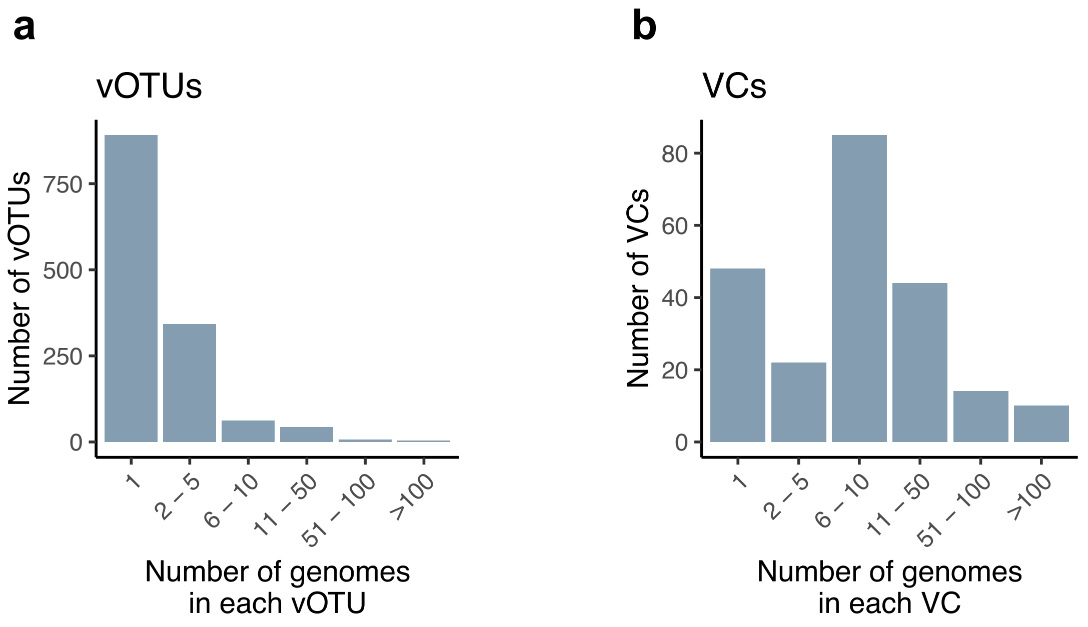

Supplementary fig. 2 | Statistics of vOTU and VC construction
The number of vOTUs and the number of phage genomes included within them.

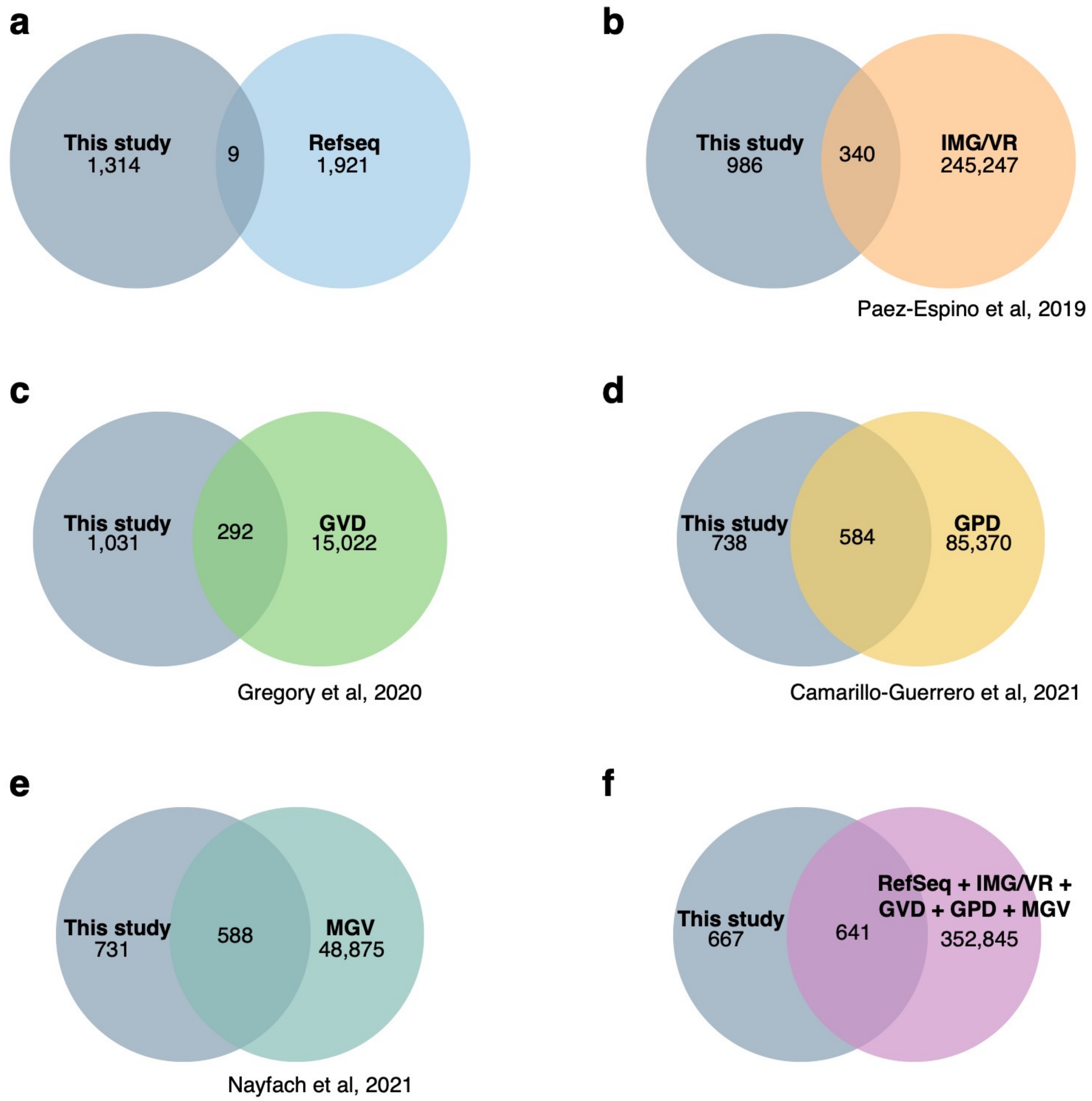

Supplementary fig. 3 | Comparison of the phage genomes discovered in this study and those in databases

Venn diagrams showing the overlaps between the phage genomes identified in this study and those in public databases. Phage genomes were clustered with >95% identity and >85% length coverage thresholds. Abbreviations: IMG/VR, integrated microbial genomes / viral resource (Paez-Espino et al, 2019); GVD, gut virome database (Gregory et al, 2020); GPD, gut phage database (Camarillo-Guerrero et al, 2021); MGV, metagenomic gut virus (Nayfach et al, 2021).

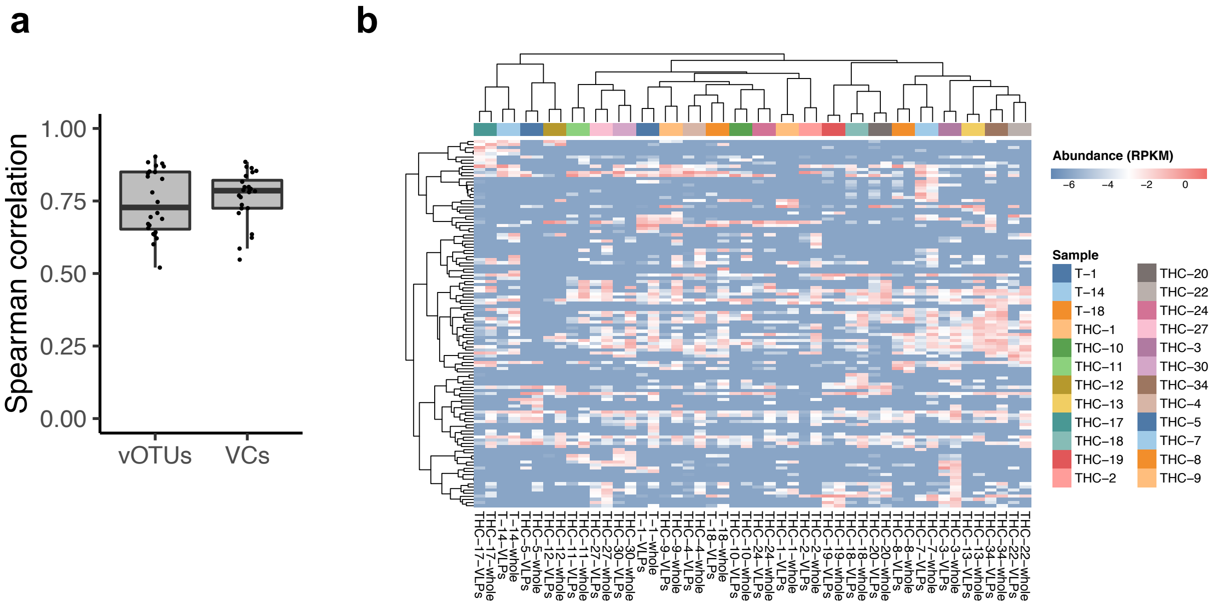

Supplementary fig. 4 | Consistencies of the viral profiles obtained from VLPs and whole metagenomes
a, Boxplot showing Spearman correlations of viral profiles between VLPs and whole metagenomes from the same fecal sample (*n* = 24). **b**, Heatmap representing viral profiles for the samples at the VC level. Red and blue colors depict high and low abundance of the VC ($\log_{10}$-transformed), respectively. Hierarchical clustering was created using the ward.D2 method based on correlations as distance. In boxplots, boxes represent the interquartile range (IQR), and the lines inside show the median. Whiskers denote the lowest and highest values within 1.5 times the IQR.

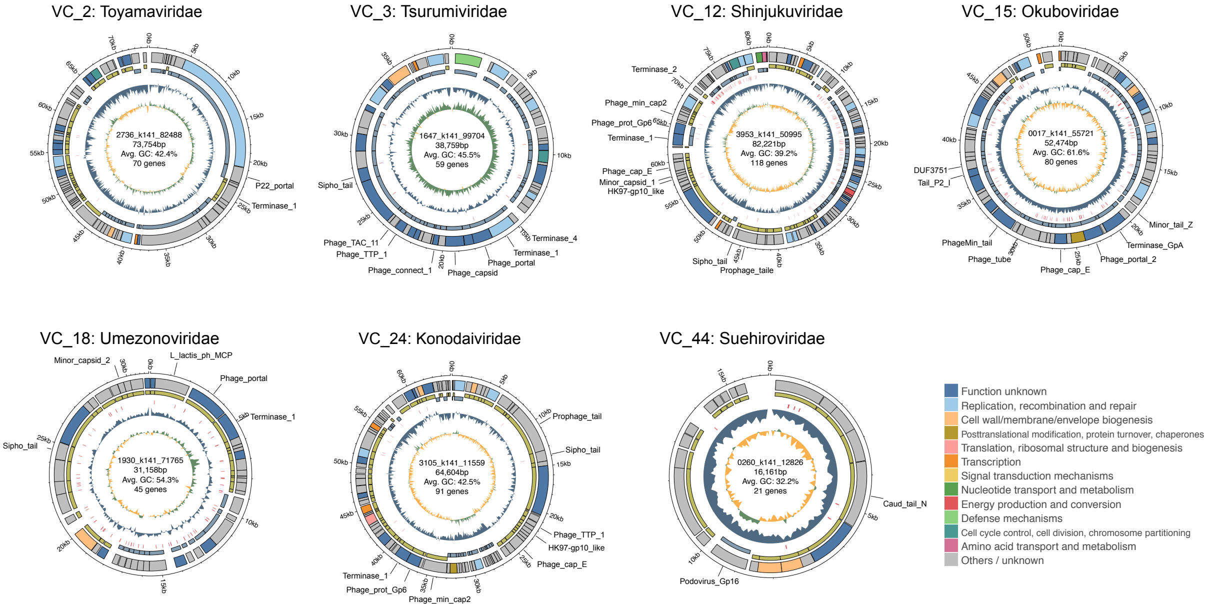

Supplementary fig. 5 | Genome structures of the seven novel major VCs

Genome structures of the seven newly identified VCs. For each VC, the representative genome of the vOTU with the highest number of contigs was selected. From inner to outer, the circles represent GC skew (green for positive and orange for negative), GC content, hit to CRISPR spacer, strand of encoded genes (blue and yellow for positive and negative strand, respectively), and functional category of the genes annotated by the eggNOG mapper. Annotation of the VHGs is shown on the genes.

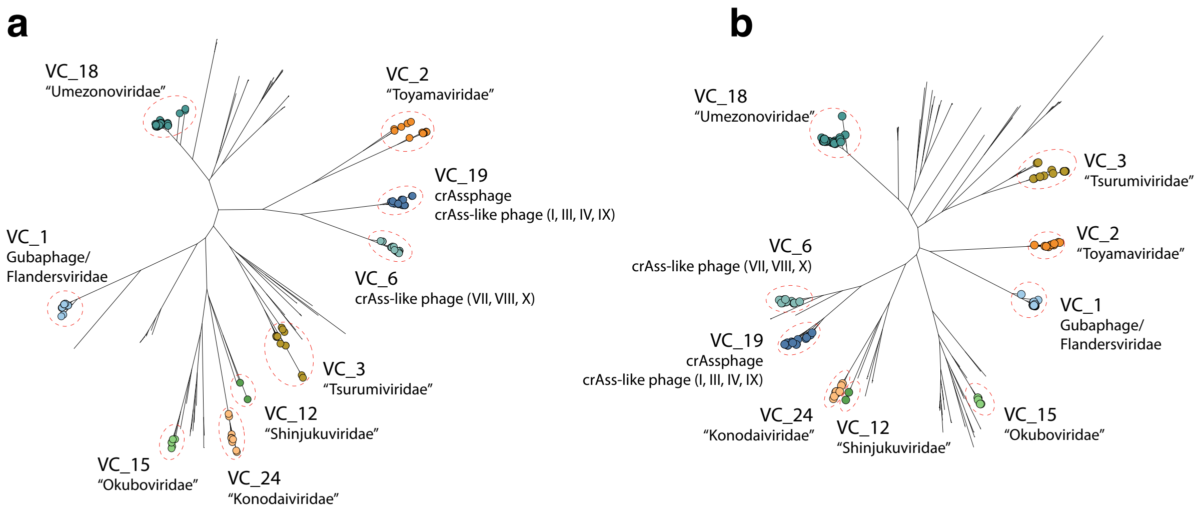

Supplementary fig. 6 | Phylogenetic relationship of the abundant VCs
Phylogenetic tree of the 10 VCs as well as reference phages in the RefSeq database based on portal (**a**) and major capsid proteins (**b**). Circles on the edges show vOTUs belonging to the VC, and edges without circles represent reference genomes in the RefSeq. Numbers in parentheses indicate candidate genera of crAss-like phages. Portal and major capsid proteins were not detected in VC_44, so the VC is not included among the trees.

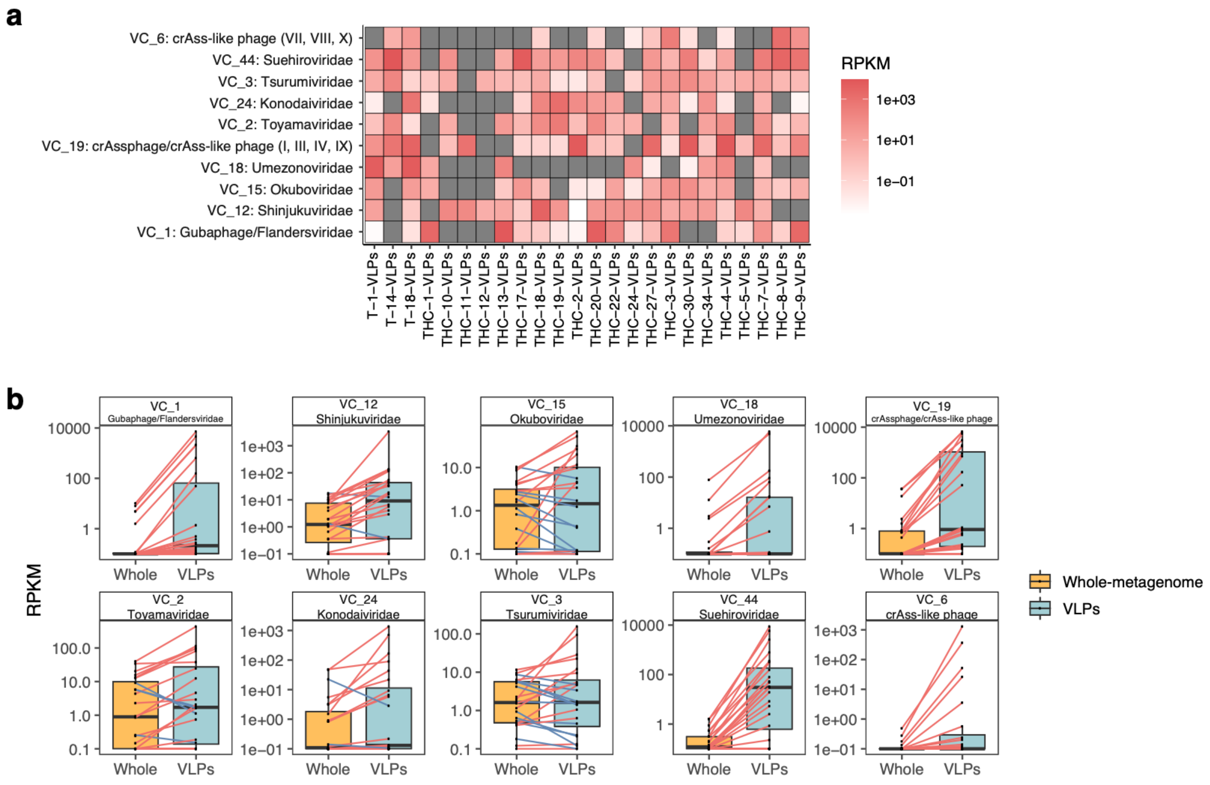

Supplementary fig. 7 | Detection of abundant VCs in the VLP metagenomes

a, Heatmap summarizing RPKM of the 10 major VCs in the 24 VLP datasets. Red and grey colors represent non-zero and zero values of RPKM, respectively. Numbers in parentheses indicate candidate genera of crAss-like phages. **b**, Comparison of RPKM for the 10 VCs between pairs of VLPs and whole metagenomes (n = 24). Each dot represents an individual sample, and those from the same faecal samples are connected with lines. Red and blue line colors show an increase or decrease of RPKM, respectively, in a VLP dataset compared with a whole metagenome. In boxplots, boxes represent the interquartile range (IQR), and the lines inside show the median. Whiskers denote the lowest and highest values within 1.5 times the IQR.

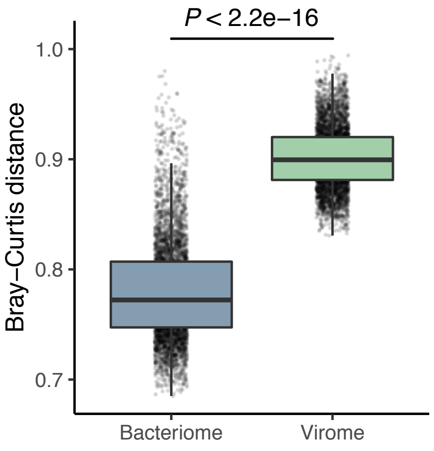

Supplementary fig. 8 | The gut virome shows higher β-diversity than the bacteriome
Boxplot comparing β-diversities (Bray-Curtis distance) of the gut virome and bacteriome Each dot represents the average value of the Bray-Curtis distance against other individuals. vOTU level and species level profiles of the virome and bacteriome were used, respectively. P-value was obtained by the Wilcoxon rank-sum test (two-sided). In boxplots, boxes represent the interquartile range (IQR), and the lines inside show the median. Whiskers denote the lowest and highest values within 1.5 times the IQR.

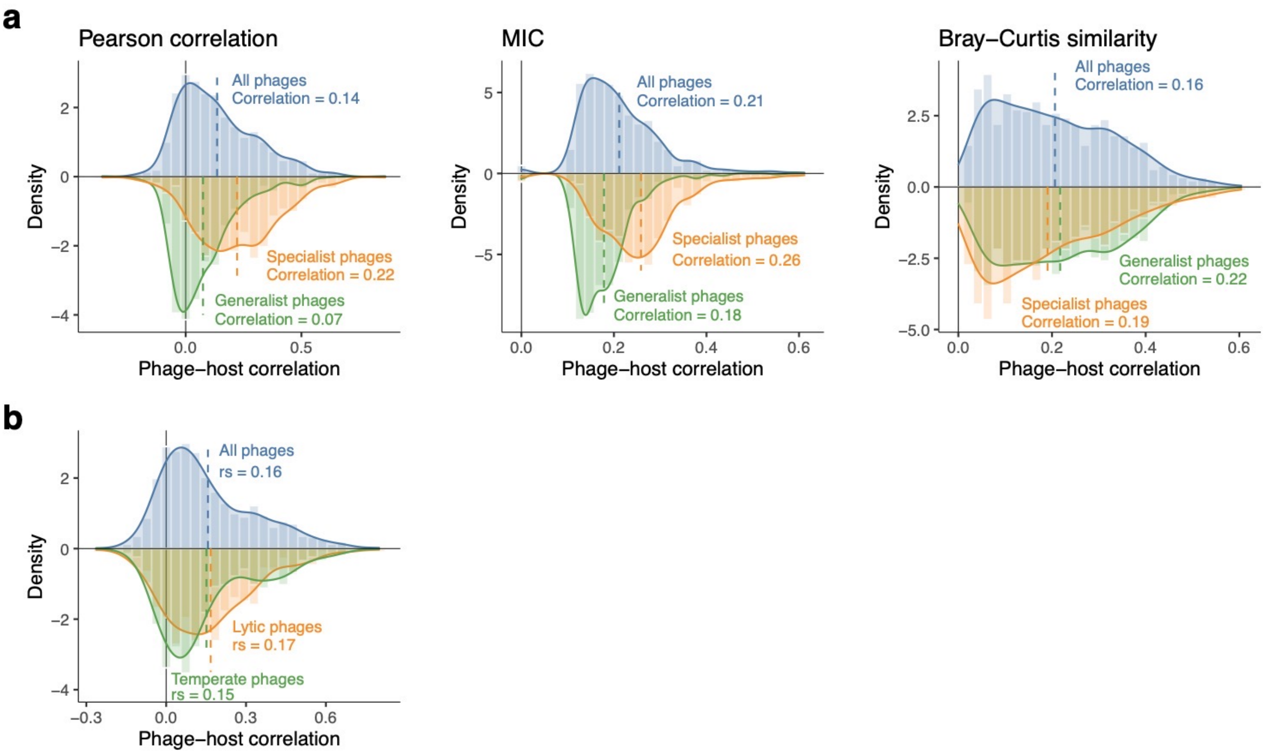

Supplementary fig. 9 | Comparison of phage-host correlations between specialist and generalist phages and between lytic and temperate phages

a, Comparison of distribution of phage-host correlations between specialist and generalist phages using different correlation indexes. Blue, orange, and green colors represent the distributions of all phages, specialist phages, and generalist phages, respectively. **b**, Comparison of distribution of phage-host correlations between lytic and temperate phages using Spearman correlation. Blue, orange, and green colors represent the distributions of all phages, lytic phages, and temperate phages, respectively. A Dashed line shows the average correlation in each distribution.

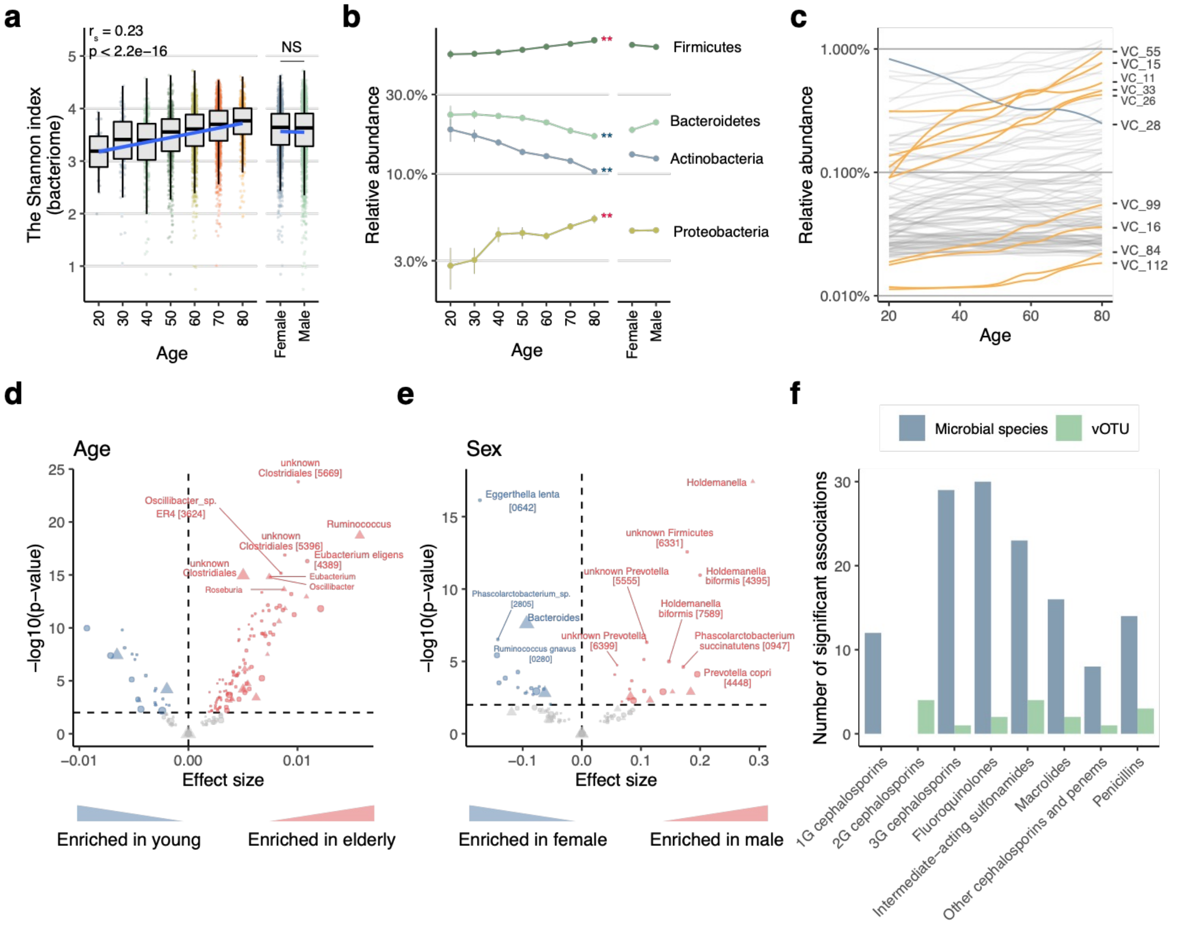

Supplementary fig. 10 | Age- and sex-related changes of the human gut virome and bacteriome

a, Correlation between age/sex and the Shannon index of the bacteriome. Individuals younger than 20 years (n = 2) and older than 80 years (n = 6) were excluded from the plot due to the low numbers of individuals. **b**, Average relative abundance of the phylum-level taxonomy of bacterial species. Red and blue asterisks show significant positive and negative correlations, respectively (* FDR < 0.01, ** FDR < 0.001). Error bars represent standard erros. **c**, Regression lines for relative abundance of each VC across ages. Orange, blue, and gray colors represent significantly increased, decreased, and non-significant VCs, respectively. 112 VCs with average relative abundance > 0.1% were included in this analysis. The regression lines were obtained by the loess method. **d**, **e**, Volcano plots showing significant associations between the bacteriome profile and age (**d**) and sex (**e**). Red, blue, and gray colors represent significant enrichment, depletion, and non-significance, respectively. The circles and triangles in the plots represent species and genus level taxonomies, respectively. Names of the top 15 most significant taxonomies are represented in the plots. **f**, Bar plots showing the number of significant associations of antimicrobial and antiviral drugs with bacterial species (blue) and vOTUs (green). The associations were detected by multivariate regression analysis adjusting for other factors. In boxplots, boxes represent the interquartile range (IQR), and the lines inside show the median. Whiskers denote the lowest and highest values within 1.5 times the IQR.

Supplementary Note 1

Comparison of phage catalogue between our pipeline and VIBRANT

To investigate how a virus-detection pipeline affects the viral coverage of the catalogue, we constructed another phage genome catalogue using VIBRANT[1] from the same metagenomic dataset ($n = 4{,}198$). The detected phage genomes by VIBRANT (high or medium quality) were filtered by checkV (>70% completeness and <10% contamination) which was comparable to the thresholds used in our custom pipeline to perform fair comparison. In total, 6,336 phage genomes were obtained using VIBRANT. When we mapped the VLP reads to the VIBRANT-based catalogue, we found that 58% of the reads were mapped, on average, which was only 1% higher than the value based on our catalogue (57.1%) even though the VIBRANT-based catalogue was composed of approximately 1.3 times more genomes than our catalogue (4,709 genomes).

Although the mapped ratio of the VLP reads to the VIBRANT-based catalogue was comparable to that based on our pipeline, we found more possible non-phage sequences in the VIBRANT-based catalogue. This result suggested that our catalogue covers approximately the same amount of dsDNA phages as catalogues from the other pipelines, while it was less contaminated by non-phage sequences.

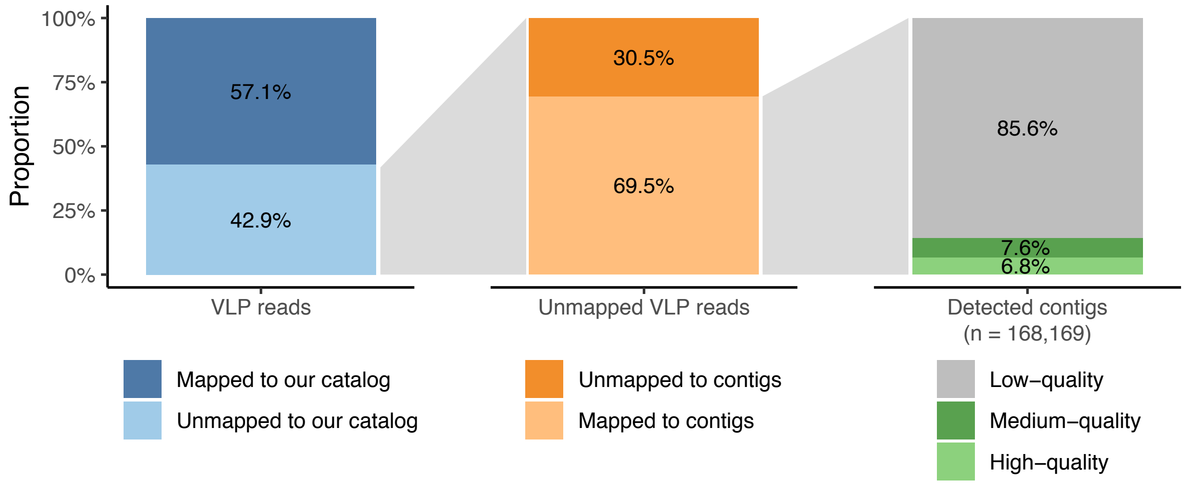

Unmapped VLP reads derived from fragmented phage contigs

To explore where the unmapped VLP reads to our catalogue come from, we constructed a non-redundant set of contigs of 4,198 whole metagenomic dataset and re-mapped the unmapped VLP reads to it. We found that 69.5 ± 21.3% (mean ± s.d.) of the unmapped VLP reads on

average were mapped to the contigs (Figure below). To characterize these possible phage contigs (n = 168,169), we performed a quality assessment by checkV[2] and found that 6.8%, 7.6% and 85.6% of the contigs were high-quality (including complete), medium-quality, and low-quality (including non-determined) phages, respectively. The majority of the contigs was low-quality, suggesting that these contigs had low abundance or repetitive regions in the genomes prevented assembly[3]. On the other hand, some of the contigs were medium-/high-quality genomes, suggesting that they were abundant enough for the assembly, but our pipeline failed to detect them as phages. It will be necessary to construct a more accurate pipeline to detect such phages to cover more varied viral sequence spaces in the human gut.

Comparison of crAss-like phage abundance in our cohort with that reported in a previous study

The most abundant viral cluster (VC) in our cohort was crAss-phage containing one (VC_19) with a relative abundance of 4.3% on average. When we merged all relative abundances of crAss-like phage-related VCs (VC_19, VC_6 and VC_153), the total abundance was 5.2%. These results were consistent with those of previous studies indicating crAss-like phages are the most abundant phage clades in the human gut[4–7]. However, the average abundance in our cohort was substantially lower than that reported in Yutin et al[6]. which suggested the relative abundance of crAss-like phages was ~86%. This discrepancy might be derived from the difference of the reference genomes used. In our analysis, we used all of the high-quality phage genomes discovered in this study as a reference including crAss-like phages, the second most abundant Gubaphage/Flandersviridae, and the others. On the other hand, Yutin et al. seemed to use only crAss-like phage genomes and those in RefSeq[6]. Since the "relative abundance" is "the relative ratio among mapped reads", the value is dependent on the reference genomes used (i.e. the presence of the other phage clades in a reference other than crAss-phage reduces relative abundance of crAss-like phages). It will be necessary to construct a comprehensive and high-quality reference genome catalogue for the human gut phages to accurately determine the relative abundance of each phage clade.

Supplementary references

1. Kieft, K., Zhou, Z. & Anantharaman, K. VIBRANT: automated recovery, annotation and curation of microbial viruses, and evaluation of viral community function from

genomic sequences. *Microbiome* **8**, 90 (2020).

2. Nayfach, S. *et al.* CheckV assesses the quality and completeness of metagenome-assembled viral genomes. *Nat. Biotechnol.* (2020) doi:10.1038/s41587-020-00774-7.
3. Kiguchi, Y., Nishijima, S., Kumar, N., Hattori, M. & Suda, W. Long-read metagenomics of multiple displacement amplified DNA of low-biomass human gut phageomes by SACRA pre-processing chimeric reads. *DNA Res.* **28**, dsab019 (2021).
4. Dutilh, B. E. *et al.* A highly abundant bacteriophage discovered in the unknown sequences of human faecal metagenomes. *Nat. Commun.* **5**, 4498 (2014).
5. Yutin, N. *et al.* Discovery of an expansive bacteriophage family that includes the most abundant viruses from the human gut. *Nat Microbiol* **3**, 38–46 (2018).
6. Yutin, N. *et al.* Analysis of metagenome-assembled viral genomes from the human gut reveals diverse putative CrAss-like phages with unique genomic features. *Nat. Commun.* **12**, 1–11 (2021).
7. Guerin, E. *et al.* Biology and Taxonomy of crAss-like Bacteriophages, the Most Abundant Virus in the Human Gut. *Cell Host Microbe* **24**, 653–664.e6 (2018).
