# Supplementary material for: Extensive gut virome variation and its associations with host and environmental factors in a population-level cohort
Source: Nat Commun. 2022 Sep 6;13:5252. doi: 10.1038/s41467-022-32832-w (PMC9448778; doi:10.1038/s41467-022-32832-w)
Supplement: Supplementary file 3 — Description of additional supplementary files [file 41467_2022_32832_MOESM3_ESM.docx]

File Name: **Supplementary Data 1**

Description: Summary statistics of the metagenomic data

File Name: **Supplementary Data 2**

Description: List of collected metadata

File Name: **Supplementary Data 3**

Description: List of VHGs and plasmid related genes used in the viral detection pipeline

File Name: **Supplementary Data 4**

Description: Sequence statistics of the phage genomes in the catalogue

File Name: **Supplementary Data 5**

Description: Sequencing statistics of VLP and bulk DNA from 24 samples

File Name: **Supplementary Data 6**

Description: Summary statistics of vOTUs

File Name: **Supplementary Data 7**

Description: Summary information of the 10 major VCs

File Name: **Supplementary Data 8**

Description: List of anti-viral genes used in the analysis

File Name: **Supplementary Data 9**

Description: Correlation analysis between Shannon diversity of the virome and KEGG orthologies of the bacteriome

File Name: **Supplementary Data 10**

Description: Single and multivariate regression analysis between vOTUs and age/sex

File Name: **Supplementary Data 11**

Description: Single and multivariate regression analysis between VCs and age/sex
