# Supplementary material for: Extensive gut virome variation and its associations with host and environmental factors in a population-level cohort
Source: Nat Commun. 2022 Sep 6;13:5252. doi: 10.1038/s41467-022-32832-w (PMC9448778; doi:10.1038/s41467-022-32832-w)
Supplement: Supplementary file 15 — Reporting Summary [file 41467_2022_32832_MOESM15_ESM.pdf]

nature portfolio

Corresponding author(s): Naoyoshi Nagata and Suguru Nishijima

Last updated by author(s): Jul 27, 2022

Reporting Summary

Nature Portfolio wishes to improve the reproducibility of the work that we publish. This form provides structure for consistency and transparency in reporting. For further information on Nature Portfolio policies, see our Editorial Policies and the Editorial Policy Checklist.

Statistics

For all statistical analyses, confirm that the following items are present in the figure legend, table legend, main text, or Methods section.

n/a	Confirmed	
☐	☒	The exact sample size (*n*) for each experimental group/condition, given as a discrete number and unit of measurement
☐	☒	A statement on whether measurements were taken from distinct samples or whether the same sample was measured repeatedly
☐	☒	The statistical test(s) used AND whether they are one- or two-sided<br>*Only common tests should be described solely by name; describe more complex techniques in the Methods section.*
☐	☒	A description of all covariates tested
☐	☒	A description of any assumptions or corrections, such as tests of normality and adjustment for multiple comparisons
☐	☒	A full description of the statistical parameters including central tendency (e.g. means) or other basic estimates (e.g. regression coefficient) AND variation (e.g. standard deviation) or associated estimates of uncertainty (e.g. confidence intervals)
☐	☒	For null hypothesis testing, the test statistic (e.g. *F*, *t*, *r*) with confidence intervals, effect sizes, degrees of freedom and *P* value noted<br>*Give P values as exact values whenever suitable.*
☒	☐	For Bayesian analysis, information on the choice of priors and Markov chain Monte Carlo settings
☒	☐	For hierarchical and complex designs, identification of the appropriate level for tests and full reporting of outcomes
☐	☒	Estimates of effect sizes (e.g. Cohen's *d*, Pearson's *r*), indicating how they were calculated

Our web collection on statistics for biologists contains articles on many of the points above.

Software and code

Policy information about availability of computer code

Data collection	No commercial software has been used for data collection.
Data analysis	Bowtie2 (v2.2.1), MEGAHIT (v1.2.9), MetaGeneMark (v3.38), DeepVirFinder (v1.0), hhsuite (v3.2.0), pipeline_for_high_sensitive_domain_search (v0.1.3), fetchMG (v1.0), barrnap (v0.9), CheckV (v0.7), VirSorter (v1.0.3), Virsorter2 (v2.2.3), VIBRANT (v1.2.1), Seeker (v1.0.3), ViralVerify (v1.1),, dRep (v2.2.3), MCL (v14-137), PILER-CR (v1.06), MiniMap2 (2.15-r905), MAFFT (v7.458), Trimal (v1.4rev15), FastTree (2.1.10), iTOL, eggNOG-mapper (v1), DIAMOND (v0.9.24.125), mOTUs2 (v2.1.1), fastp (v0.20.1), iTOL (v5), R (v3.6.1), R packages glm2 (v1.2.1), vegan (v2.5.7), iNEXT (v2.0.20), Rtsne (v0.16), and circlize (v0.4.15). Detailes are described in the methods section.<br><br>The custom phage-detection pipeline used in this study is available at https://gitlab.com/suguru.nishijima/phage_detection.

For manuscripts utilizing custom algorithms or software that are central to the research but not yet described in published literature, software must be made available to editors and reviewers. We strongly encourage code deposition in a community repository (e.g. GitHub). See the Nature Portfolio guidelines for submitting code & software for further information.

Data

Policy information about availability of data

All manuscripts must include a data availability statement. This statement should provide the following information, where applicable:

- Accession codes, unique identifiers, or web links for publicly available datasets
- A description of any restrictions on data availability
- For clinical datasets or third party data, please ensure that the statement adheres to our policy

Sequence statistics of the 4,198 individuals and cohort-level summaries of the metadata are available in Supplementary Table 1 and 2, respectively. All circular and linear phage genomes detected in this study (n = 4,709) are available in the NCBI GenBank (accession number OP030729-OP031128 and OP072211-OP076519) and at https://doi.org/10.5281/zenodo.5645361.

Reference bacterial, archaeal, viral and plasmid genomes were downloaded from the RefSeq database. Genomes of human gut phage constructed in previous studies were downloaded as follows. GVD: https://datacommons.cyverse.org/browse/iplant/home/shared/iVirus/Gregory_and_Zablocki_GVD_Jul2020/. MGV: https://portal.nersc.gov/MGV/. GPD: http://ftp.ebi.ac.uk/pub/databases/metagenomics/genome_sets/gut_phage_database/. IMG/VR2: https://img.jgi.doe.gov/cgi-bin/vr/main.cgi.

Field-specific reporting

Please select the one below that is the best fit for your research. If you are not sure, read the appropriate sections before making your selection.

- [x] Life sciences
- [ ] Behavioural & social sciences
- [ ] Ecological, evolutionary & environmental sciences

For a reference copy of the document with all sections, see nature.com/documents/nr-reporting-summary-flat.pdf

Life sciences study design

All studies must disclose on these points even when the disclosure is negative.

Sample size	No sample size calculation was performed because this was exploratory observational study.
Data exclusions	Of 4,241 metagenomic samples sequenced, 43 samples were excluded due to low sequence quantities.
Replication	External validation was not conducted in this study.
Randomization	Not applicable because this study is not a randomized study.
Blinding	Taxonomic and functional analysis based on the metagenomic data were blinded to clinical information.

Reporting for specific materials, systems and methods

We require information from authors about some types of materials, experimental systems and methods used in many studies. Here, indicate whether each material, system or method listed is relevant to your study. If you are not sure if a list item applies to your research, read the appropriate section before selecting a response.

Materials & experimental systems

n/a	Involved in the study	
x		Antibodies
x		Eukaryotic cell lines
x		Palaeontology and archaeology
x		Animals and other organisms
	x	Human research participants
x		Clinical data
x		Dual use research of concern

Methods

n/a	Involved in the study	
x		ChIP-seq
x		Flow cytometry
x		MRI-based neuroimaging

Human research participants

Policy information about studies involving human research participants

Population characteristics	We collected fecal samples from 4,241 Japanese individuals and analyzed 4,198 samples (59% males, mean age 66.4), after excluding 43 samples due to low sequence quantities.
Recruitment	The Japanese 4D microbiome project is a prospective multicenter registry in Japan. Data entry commenced in January 2015 and is ongoing. Participants registered in the project are those who visit hospitals in the area for disease diagnosis or a health checkup. Fecal samples are collected from both healthy and diseased participants. The eligibility criteria for participants are as follows: (1) born and raised in Japan; (2) age > 15 years; (3) written informed consent provided; and (4) having an endoscopic diagnosis on colonoscopy for colorectal cancer screening, surveillance, and diagnosis of various gastrointestinal symptoms. The exclusion criteria were as follows: (1) suspected acute infectious disease based on clinical findings (e.g., acute enterocolitis, pneumonia, tuberculosis etc.); (2) acute bleeding; (3) hearing loss; (4) unable to understand written documents; (5) unable to write and (6) limited ability to perform activities of daily living.<br><br>Since this is a study for the gut microbiome, more participants with higher health concerns may participate (i.e. self-selection bias). In addition, there may be an observer bias because some of the disease information was collected from electronic medical records. The collector's medical knowledge could affect the proportion of certain diseases.
Ethics oversight	Before starting this study, written informed consent was obtained prior to participation in the project. The study protocol for the Japanese (Disease, Drug, Diet, Daily life) microbiome project was approved by the medical ethics committees of the Tokyo Medical University (Approval No: T2019-0119), National Center for Global Health and Medicine (Approval No: 1690), the University of Tokyo (Approval No: 2019185NI), Waseda University (Approval No: 2018-318), and the RIKEN Center for Integrative Medical Sciences (Approval No: H30-7). We conducted a prospective cross-sectional study from 4,198 individuals participating in the Japanese 4D microbiome project, which commenced in January 2015 and is ongoing.

Note that full information on the approval of the study protocol must also be provided in the manuscript.
